# Determination of Milk Products in Ceramic Vessels of Corded Ware Culture from a Late Eneolithic Burial

**DOI:** 10.3390/molecules23123247

**Published:** 2018-12-07

**Authors:** Lukáš Kučera, Jaroslav Peška, Pavel Fojtík, Petr Barták, Diana Sokolovská, Jaroslav Pavelka, Veronika Komárková, Jaromír Beneš, Lenka Polcerová, Miroslav Králík, Petr Bednář

**Affiliations:** 1 Regional Centre of Advanced Technologies and Materials, Department of Analytical Chemistry, Faculty of Science, Palacký University, 17. listopadu 12, 779 00 Olomouc, Czech Republic; lukas.kucera@upol.cz (L.K.); petr.bartak@upol.cz (P.B.); dia.sokolovska@gmail.com (D.S.); 2 Archaeological Centre Olomouc, U Hradiska 42/6, 779 00 Olomouc, Czech Republic; peska@ac-olomouc.cz; 3 Institute of Archaeological Heritage Brno, Kaloudova 1321/30, 614 00 Brno, Czech Republic; pavfojtik@seznam.cz; 4 Centre of Biology, Geoscience and Environmental Education, University of West Bohemia, Sedláčkova 15, 30614 Plzeň, Czech Republic; japetos@cbg.zcu.cz; 5 Laboratory of Archaeobotany and Palaeoecology, Faculty of Science, University of South Bohemia, Na Zlaté stoce 3, 370 05 České Budějovice, Czech Republic; verokomar@seznam.cz (V.K.); benes.jaromir@gmail.com (J.B.); 6 Laboratory of Morphology and Forensic Anthropology (LaMorFA), Department of Anthropology, Faculty of Science, Masaryk University, Kotlářská 2, 611 37 Brno, Czech Republic; polcerova@seznam.cz (L.P.); mirekkralik@seznam.cz (M.K.)

**Keywords:** ceramic vessels, laser desorption–ionization, mass spectrometry, milk, enzyme-linked immunosorbent assay, Eneolithic period, Corded Ware culture

## Abstract

In this study, a soil from two ceramic vessels belonging to Corded Ware culture, 2707–2571 B.C., found in a cremation grave discovered in Central Moravia, Czech Republic, was analyzed using matrix-assisted laser desorption/ionization–mass spectrometry (MALDI–MS) combined with advanced statistical treatment (principal component analysis, PCA, and orthogonal projection to latent structures discriminant analysis, OPLS-DA) and by enzyme-linked immunosorbent assay (ELISA). MALDI–MS revealed the presence of triacylglycerols in both vessels. This analytical technique was used for the analysis of the soil content from archaeological ceramic vessels for the first time. Targeted ELISA experiments consequently proved the presence of milk proteins in both ceramic vessels. These results represent the first direct evidence of the use of milk or dairy products in the Eneolithic period in Moravian Corded Ware Culture and help to better understand the diet habits and living conditions of Eneolithic populations in Central Europe.

## 1. Introduction

The analysis of human cremations from archaeological burials is a very important part of archaeological research that provides valuable information for a better understanding of former populations’ habits. However, this research is difficult in general, especially because of a high level of bone fragmentation, size changes, thermal fractures and distortions, and possible artificial changes of bones related to burial rite habits [1,2,3,4]. In such a situation, explicit sex assessment, age estimation at death, population affinity, body size estimations, etc. are almost impossible. However, the obtained information can be completed by a detailed chemical characterization of graves’ content accompanying human remains (i.e., ceramic vessels containing certain material, residues of food, personal things, gifts, etc.).

Analysis of lipid residues present in ceramic vessels has already provided information about vessels’ usage and former content. Analysis of isotope ratios of individual fatty acids adsorbed in prehistoric and medieval ceramics using gas chromatography–combustion–isotope ratio mass spectrometry (GC–C–IRMS) appeared to be an effective tool for the identification of fat origin [5,6]. The detection of milk residues in archaeological contexts was already described by many authors [7,8,9,10]. The oldest evidence of storage of milk products in pottery was described by Evershed et al. [11] (by using GC–C–IRMS). These authors analyzed more than 2000 archaeological samples from Near East and Southeastern Europe, and milk lipids were detected in a wide range of historical periods, from the seventh millennium B.C. The presence of milk lipids in ceramic vessels from the Neolithic period indicates milking skills and usage of dairy products in this period. Compared with the published studies dealing with the analysis of organic residues of milk adherent to the surface of a ceramic vessel or soaked into ceramics from Copper Age (Turkey [12]), Bronze Age (England [7]), and Iron Age sites (Netherlands [13], Scotland [9]), more ceramic vessels or shreds containing milk are found from the Neolithic period, e.g., ceramic vessels from Sweden [14], Anatolia [15], Slovenia [16], France [17], Switzerland [18], England [19,20], Germany, and Italy [21,22]. GC–C–IRMS is the method mainly used. However, there are some papers dealing with the detection of milk lipids in archaeological ceramics by direct temperature–resolved mass spectrometry and nanoelectrospray mass spectrometry [13,17]. Matrix-assisted laser desorption/ionization–mass spectrometry (MALDI–MS) has been used for the study of milk residues mainly in paintings, especially for the analysis of proteins after trypsin digestion and lipids [23,24,25,26,27,28,29,30]. Besides, MALDI–MS was used for proteomic analysis of a compact organic residue found inside a more than 4000-year-old container [7].

Another promising possibility for the exact determination of organic matrices (i.e., milk, etc.) is the utilization of immunological tests. In archaeological samples, thermostable native as well as modified (partially degraded) proteins are found. On the other hand, many factors limit antibody tests applicability when dealing with archaeological material. Possible cross-reactions [31,32], degradation of proteins resulting in antigen binding—nonspecific reactions in ELISA tests [33], and contamination from surrounding areas are the main risks that need to be kept in mind when analyzing non-collagenous proteins from human and other mammal samples of modern and ancient origin [34]. Nevertheless, following a proper experimental design including sufficient reference samples and a reasonable amount of native proteins in the organic residues under study, the commercially available ELISA kits can be used with an acceptable degree of reliability. This has been already shown by the analysis of animal proteins in Neolithic samples (i.e., detection of heat-stable species-specific muscle proteins) [35]. Besides, it is possible to utilize antibody tests targeted specifically to denatured proteins that provide reliable results with a high selectivity. A wide range of detection tests based on an immunological reaction that were developed for the identification of various components in heat-treated foods are commercially available today. This category of commercial tests is purposefully designed to work with degraded traces of biological tissues in the food industry and they are undergoing a rigorous evaluation of their ability to correctly detect the ingredients in processed food [36]. It can be emphasized that their suitability for archaeology has been already proven by the identification of damaged and denatured proteins in desiccated and partially carbonized organic residues from antiquity [37].

The usage of milk by prehistoric populations is problematic and still not well explained. Analysis of ancient DNA (aDNA) shows that almost all people at the time (i.e., populations living in (E)Neolith) failed to digest lactose in adult age [38,39,40]. Currently, in Europe, only a single mutation of the lactase gene, called 13910 * T, allowing to digest lactose in adulthood was found. This supports the idea of the spreading of this mutation from one area [41]. The 13910 * T mutation was mainly detected in samples from the Late Bronze Age and Hallstatt period [38,39,40]. The mutant allele allowing lactose digestion occurred in Europe even before the Bronze Age, but its geographical distribution is uneven (e.g., in Scandinavia, where the hunter–gatherer economy prevailed, the mutant allele’s frequency in the population was 5% in the analyzed assemblage). The prevalence of the mutant allele allowing the digestion of lactose in contemporary humans is about 35%, and, in some North European populations, it is up to 90% in adults [42]. The milk of dairy animals as a complex admixture of many important nutrients—proteins, fats, minerals, vitamins, and others [43]—is a nutritionally beneficial food and after natural fermentation (without any complex cultural addition) it can be digested even by lactase activity-deficient people [44]. Based on the above-mentioned evidence, we hypothesize that milk served as food in Neolithic–Eneolithic Europe, but not in fresh form. To decrease the amount of lactose, milk processing could be used, i.e., fermentation and/or heating [44]. Therefore, molecular genetic event(s) in the evolution of lactase persistence in adults might be a secondary by-product of herding dairy animals. Anthropological reconstructions [45] showed that dairy product consumption and adoption by human cultures preceded the evolution of lactase persistency by thousands of years (milking came first, lactose digestion followed) [46]. Additionally, using milk in nutrition might have been important not only for nutritional purposes. For example, the fermented milk products might have positive health effects to gut microbiota during nomadic movements over long distances and in diverse environments [47]. Therefore, the direct detection of milk proteins and lipids in archeologically excavated vessels might be, in our opinion, a much better indicator of the time in prehistory of dairy product adoption for human persistence and economic strategies than any molecular genetic estimations of the evolution of lactase persistence.

In this contribution, the content of two archaeological ceramic vessels from a grave belonging to the Moravian Corded Ware culture was analyzed by matrix-assisted laser desorption/ionization–mass spectrometry (MALDI–MS) and enzyme-linked immunosorbent analysis (ELISA). To the best of our knowledge, this is the first time that MALDI–MS is used for the analysis of ancient milk fat residues from the soil content of ceramic vessels. Lipid profiling by MALDI–MS revealed the presence of milk residues in the bottom soil layers of the excavated vessels. The subsequent ELISA experiments confirmed the occurrence of milk proteins. Both methods thus mutually confirmed the presence of milk or dairy products in the investigated vessels. Information about the utilization of milk products in the Eneolithic period in Central Europe is of key importance for the description of the Eurasian population’s migration at that time and the expansion of lactase gene mutation.

## 2. Results

**Figure 1:**
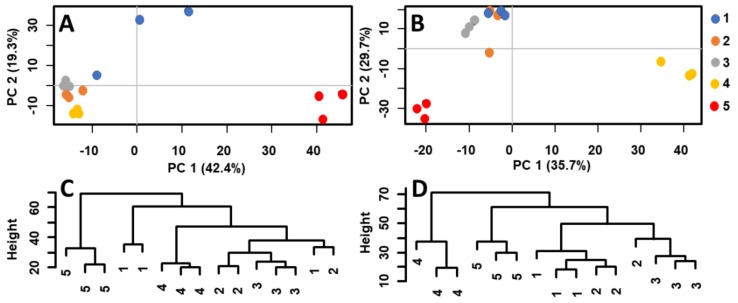
Score plots and dendrograms of MALDI-MS data measured in acetone extracts of five soil layers from ceramic vessels no. 4 (**A,C**) and no. 5 (**B,D**), respectively.

The soil extracts were analyzed by MALDI–MS, and the raw data were transferred to a statistical software and studied by PCA, HCA, and OPLS-DA. Figure 1 shows the score plots (PCA) of MALDI–MS data obtained by the analysis of acetone extracts of separated soil layers (for details see Experimental, Chapt. 3.2). A distinct segregation of the bottom samples (5th layer) from the upper ones (1st–3rd layer; 4th layer not considered) was observed in ceramic vessel no. 4. Each sample was measured in three chemical replications represented by three points of particular color (Figure 1A). A similar pattern could be observed in the soil extracts taken from vessel no. 5 (Figure 1B). Here, the 4th and 5th layer significantly segregated from the upper layers. Hierarchical clustering was used to study the similarity of specific layers. Figure 1C,D shows the dendrograms of the relationships between the soil extracts of ceramic vessels no. 4 and 5. Generally, the soil samples from the 4th and 5th layers of both ceramic vessels were located in separated clades (i.e., leaves 4 and 5 were dissimilar to other leaves, with the exemption of two measurements of layer 1 in vessel No. 4 that were probably due to a higher data dispersion visible in the related PCA plot). Both multivariate methods pointed out a significantly different composition of the bottom layer(s). MALDI–MS combined with multivariate statistics was thus a sufficiently selective tool to differentiate particular samples of soil based on variances in chemical composition. The first two components explained 61.7% (ceramic vessel no. 4) and 65.4% (ceramic vessel no. 5) of the variance in the data. Note that cumulative proportion of variance with third and fourth component explained 75.5% and 81.0% of the variance for ceramic vessel no. 4 and 75.5% and 79.9% of the variance for ceramic vessel no. 5, respectively (Supplement 4).

**Figure 2:**
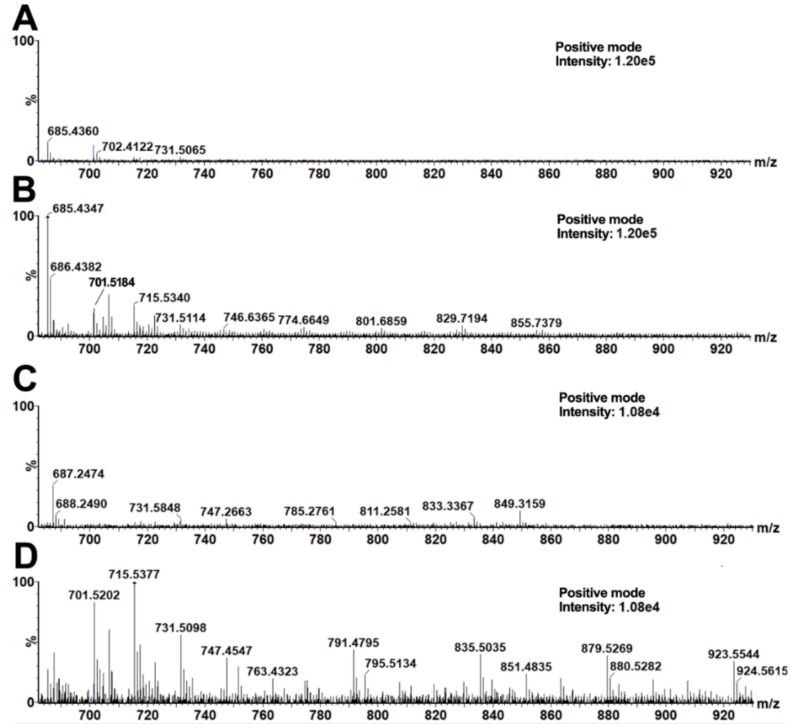
MALDI–MS spectra of acetone soil extracts from ceramic vessels no.4 (**A**,**B**) and no. 5 (**C**,**D**). The presented spectra represent one measurement of the 1st (**A**,**C**) and 5th (**B**,**D**) layers.

The following analysis of the data by OPLS-DA provided signals of significant markers describing the main differences between the upper and the bottom layers of soil in the vessels. Markers (i.e., compounds present in the bottom layer in significantly higher amounts than in the upper layer) with the highest variability and reliability at the same time were taken from a low-risk region of an appropriate S-plot (Supplement 5). For our purposes, the low-risk region was defined as a box with the following coordinates: p[1] = 30–100% and p[2] = 75–100% from the highest value on the *x*-axis and *y*-axis, respectively, as already reported [48]. Table 1 shows significant markers of the bottom soil layers taken from ceramic vessels no. 4 and no. 5. A manual inspection of appropriate MALDI mass spectra supported the significant differences in those signals, evidencing the functionality of the used OPLS-DA method. The most significant signals (taken from the appropriate S-plot) were observed in soil from pottery no. 5. (soil from the fifth, bottom layer compared to the first, upper layer). The differences between two adjacent signals corresponded to the CH_2_ group homological increments (i.e., Δ*m*/*z*(1) = 687.4951–673.4808 = 14.0143; Δ*m*/*z*(2) = 14.0163; Δ*m*/*z*(3) = 14.0172) and oxygen (Δ*m*/*z*(4) = 15.9721). These signals corresponded to potassium adducts of triacylglycerols (TAGs). A good agreement of the theoretical mass of the proposed elemental compositions with the measured values was observed. Picariello, Sacchi, and Addeo studied TAGs in various (recent) animal fats (lard, tallow, and milk fat). Particular signals of TAGs were observed in the form of sodium adducts solely in the bovine milk sample and not in the other fatty materials (i.e., *m*/*z* 657.5, 685.5, 699.6, 715.6), and, after recalculation of the sodium adducts *m*/*z* values to the corresponding potassium ones, a good agreement with our signals was observed [49]. On the basis of this agreement, the markers found in the bottom soil layers of both ceramic vessels were attributed to organic residues of dairy products. Similar signals were observed in the bottom layer of the vessel no. 4. Note that other (higher) TAGs were also found in the spectra from ceramic vessel no. 4, i.e., *m*/*z* 829.7618, 855.7787, 925.7029, 939.7148, 953.7241, 967.7419, and from ceramic vessel no. 5, i.e., 829.7870, 835.5690, 879.5928, 923.6188, 951.6760, 967.6469 (Figure 2). Note that m/z values of statistically significant triacylglycerols (TAGs) discussed here represent the average values taken from three MS spectra (repeated measurements).

The presence of fat residues in both vessels was further confirmed by targeted immunochemical tests. Results of specific ELISA tests on native β-Lactoglobulin (Table 2) confirmed the presence of milk protein residues in the bottom soil layer of vessel no. 5. A significantly positive reaction was observed (dual wave data, dwd: the difference at the two wavelengths of 450 and 650 nm was 0.044; the dwd of the negative control was 1.320). On the other hand, β-Lactoglobulin was not found in the soil from the bottom of vessel no. 4 (values approaching to negative control and dwd 1.812 were obtained). The presence of dairy products was also tested by casein ELISA kit. A positive reaction was observed in both studied vessels. Note that a weaker positive reaction was also observed in the reference soil sample from the surrounding area. Values of 0.13 and 0.17 (i.e., close to the value of 0.2 corresponding to 0.5 mg/kg of casein standard) were obtained that were significantly lower than the value determined for both vessels. These data confirmed the presence of dairy products in both ceramic vessels. The usage of dairy products in ancient diet has already been proved by many authors (analysis of organic residues of dairy products adherent to a surface of a ceramic vessel or soaked into ceramics) [15,16,21]. It can be emphasized that this is the first application of MALDI–MS to analyze ancient milk fat residues in the soil content of ceramic vessels.

Although milk was part of ancient diet, it is questionable whether populations in Eneolith were already able to digest lactose (milk sugar). Recent findings from Hungary focused on the Iron Age show a possible milk consumption by nomads, based on the proven genetic proximity of the Hungarian population to eastern populations [39]. Nomads from the Late Bronze and Iron Age living in this region usually belonged to the Indo-European historical Cimmerians [50]. However, it is still unknown when and where the European mutation arose. In this sense, the presence of bovine milk products from the end of the Eneolithic period in Moravia discussed and confirmed in this paper is extraordinarily important and evokes the need of a more proper genetic research of ancient remains. 

## 3. Materials and Methods

### 3.1. Archaeological Description of the Inspected Cremation Grave

**Figure 3:**
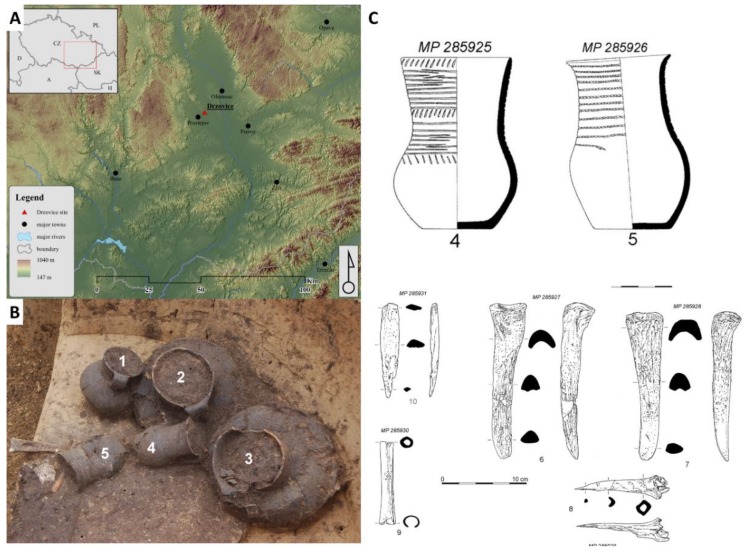
Place of rescue excavation (**A**), five ceramic vessels in the grave (**B**), and drawing of ceramic potteries and bone tools (**C**).

The investigated cremation grave was discovered during a rescue excavation close to the village of Držovice (Prostějov, Central Moravia, Czech Republic, Figure 3) in the years 2014–2015. Five graves were found at the burial ground. The soil from two ceramic vessels coming from a grave denoted H4 was analyzed. The detailed archaeological description of the excavation close to the village of Držovice is presented in a paper by Fojtík [51]. The two ceramic vessels were described as a pair of Corded Ware beakers (ceramic vessel no.4 and no.5 with volumes of 0.8 and 0.6 L, respectively) with imprints of cord (circumferential grooves and a combination of inclined cuts on the neck). Beside the studied vessels, bone industries represented by a pair of massive chisels (radius or metapodium? *Bos taurus*, Supplement 1), a bone tip and awl (shinbone of medium-large mammal, presumably from sheep or goat, Supplement 2), and a worked tube bone (radius of sheep or goat, Supplement 2) were found in the grave. The presence of those bones illustrates the circumstances of the funeral, and they are described in more details.

The cord beakers with a sigmoid profile and the classic form of a jug of Dřevohostice type (CD1: variant Dřevohostice) belong to the earliest period of the Moravian Corded Ware culture (MCW; phase IIIa). The exact dating was performed by the radiocarbon method (Beta Analytic Radiocarbon Dating, Miami, USA) on a bone chisel found in the grave. The high-Probability Density Range Method, Intcal 13 was used, and the period of the bone material was determined to be in the ranges 2707–2571 B.C. (probability, prob = 62.3%), 2863–2807 (prob = 22%), 2759–2717 (prob = 9.9%), and 2513–2503 (prob = 1.1%). These data confirm that the bone chisel and consequently the grave belong to Eneolithic period. A particularly interesting item in the grave is a small bony tube (length: 94.75 mm; width 14.02 mm; outer diameter 14–15 mm, inner diameter 7.45–8.92 mm). This type of artefacts was found in Central Europe in the tombs of several groups of Corded Ware culture. Bony tubes could come from the earlier Eneolithic cultures in southern Ukraine and Pribajkali. The recent finding of a damaged and perhaps incomplete bone tube with a hint of engraved decoration in the late Jevišovice culture settlement at Kroměříž 3-Miňůvky could confirm the relationships of this tube to East (South-East) Europe.

The anthropological analysis followed the already published recommendations for the assessment of human cremated remains [52,53,54]. Most of the fragments ranged in color from light grey to white and were classified accordingly into the category of burning temperature 900 °C or higher [55]. Preserved parts of skulls indicated that the burial content represented remains of (at least) two human individuals, which was also supported by the weight of the bone fragments (2,146.6 g) [53,56]. One preserved third molar indicated that the age at death of one of the individuals was at least 18 years [57]. Skeletal fragments (pieces larger than 2 mm) were measured by means of the original semiautomatic metric procedure developed by Polcerová [58,59]. The lateral angle values measured in the preserved pyramid of the temporal bone ranged from 54° to 62°, which is in the zone of variation occupied predominantly by females in the reference sample [60]. To sum up, the grave H4 represented remains which were well burned during regular cremation and contained skeletal and teeth fragments of two or more humans of whom at least one was an adult and at least one was a female (for more details see Supplement 3). 

### 3.2. Sample Preparation

The ceramic vessels were compact but with visible cracks. The sherds were gently removed, and the internal part of the ceramic vessel (soil) that remained in a compact piece with the shape of the vessel was carefully reinforced by food plastic foil and transported to the laboratory. The soil material from the ceramic vessels was divided manually by a big knife in the vertical direction into five equal parts. Each part was consecutively extracted with four solvents with different polarity and acidity, i.e., water, 0.05% ammonium hydroxide in methanol, 1% formic acid in methanol, and acetone, providing the extraction of a wide number of chemical structures. All chemicals were purchased from Penta Ltd. (Prague, Czech Republic). The individual extracts were filtered through cellulose filter paper (black label, pore size 7–8 µm, Schleicher & Schuell A.G., Feldmeilen, Switzerland) and concentrated to a defined volume of 1 mL using a fine stream of nitrogen. The aqueous extracts were concentrated by lyophilization to the same volume. The concentrated extracts were subjected to analysis by MALDI–MS (experimental parameters are given below).

### 3.3. Matrix-Assisted Laser Desorption/Ionization–Mass Spectrometry and Multivariate Data Analysis

MALDI–mass spectrometry was used for a non-targeted analysis of the extracts of the soil content of both vessels. A Synapt G2-S high-resolution tandem mass spectrometer (Waters, Milford, MA, USA) with a vacuum MALDI source and a hybrid QqTOF type of mass analyzer with integrated two collision cells and one ion mobility cell was used. Two microliters of sample extract were applied on MALDI target plate, and after solvent evaporation, the formed residue was covered by the matrix solution. As matrix, 2,4,6-trihydroxyacetophenone (THAP, Sigma-Aldrich, St. Louis, USA, dissolved in acetonitrile/water (1:1, *v*/*v*), at a concentration of 25 mg/mL) was used. This matrix is appropriate for lipid analysis by MALDI–MS technique [61]. The following parameters were chosen for of MALDI–MS: Mass range: 50–2000 Da, ionization mode: positive, MALDI extraction voltage: 10 V, hexapole bias: 10 V, laser: 1 kHz, Nd:YAG solid state, 355 nm wavelength with variable pulse energy, 50 μJ @ 1000 Hz, pulse width 2 ns, peak power 25 kW, laser energy: 450 (arb). MassLynx 4.1 (Waters) software was used for data collection.

The obtained MALDI–MS data were processed by MarkerLynx XS software, optional part of MassLynx, allowing extraction, normalization, and alignment of *m*/*z* values and intensities (formation of data matrix from raw MALDI–MS spectra). The method parameters were set as follows: Analysis type: Combined scan range, peak separation: 0.05 Da, marker intensity threshold: 1000 counts.

Logarithmic transformation was applied. The transformed data matrix was transferred to freeware environment for statistical computing R-project, version 3.5.0 [62] and studied by principal component analysis (PCA) and orthogonal projections to latent structures discriminant analysis (OPLS-DA) (package “muma” [63]). Pareto scaling was used. The outputs were evaluated in the form of appropriate biplots and S-plots. For hierarchical clustering analysis (HCA), the default package “stats” was used. The complete linkage algorithm was applied on the data [62].

### 3.4. Enzyme-Linked Immunosorbent Analysis

The soil samples from the studied Eneolithic ceramic vessels were tested for the presence of dairy products from cattle. Tests were made using BIOKITS BLG (β-Lactoglobulin, βLG) Assay kit (Neogen Corporation, MI, USA) and R4612 RIDASCREEN Fast Casein (R Biopharm AG, Germany). We used the competitive enzyme-linked immunosorbent assay (ELISA) test. In total, 2 mg of crushed and grinded sample was mixed with 150 ml of extraction buffer (Allergen extraction buffer without additive, concentration 1:10), mixed vigorously using a vortex, and put for 10 min into a water bath thermostated at 60 °C. After incubation, the sample was cooled down (ice water) and centrifuged for 5 s at low speed (1000 RPM). Then, 100 µL of supernatant was used for the ELISA test. The procedure followed the protocol suggested by the kit producer [64]. βLG-biotin was added to the extract solution as the secondary antibody [38]. Measurements were done on a microtiter plate using ELISA competitive test. Negative reactions were revealed by a specific color (bright yellow). The labelled antigen competed for the primary antibody binding sites with the sample antigen (unlabeled). The samples were measured using an ELISA reader VERSAmax™ (Molecular Devices, San Jose, CA, USA) at 450 nm.

## 4. Conclusions

Matrix-assisted laser desorption/ionization–mass spectrometry combined with multivariate statistics revealed signals belonging to triacylglycerols in two ceramic Corded Ware beakers with volumes of 0.8 and 0.6 L. Those signals corresponded well with milk fat components formerly found in recent samples. This is the first application of matrix-assisted laser desorption/ionization–mass spectrometry for the analysis of milk fatty residues in ancient ceramic vessels. The enzyme-linked immunosorbent assay proved that the fats in both ceramic vessels originated from dairy products (i.e., positive reaction with casein). Moreover, the ELISA analysis of the soil sample from one Corded Ware beaker (0.6 L) provided a positive response in the β-Lactoglobulin assay. We can suppose that ceramic vessel no. 5 served for storage of bovine milk or dairy products. These results represent the first direct evidence of the utilization of milk products in the Eneolithic period in Moravian Corded Ware culture. Our data related to the utilization of milk in the Early Neolithic–Eneolithic period in Easter Central Europe (the Central Danube Region) significantly extend the former evidence of dairy products usage in Western Central Europe (Germany, Switzerland).

## Figures and Tables

**Table 1:** List of the most significant markers of the bottom soil layers taken from the two studied vessels.

	*m*/*z*	CN/DB	Theoretical Formula	dtm (mDa)
Ceramic vessel no. 4	673.4879	36:2	C_39_H_70_O_6_K	−7.0
	687.5005	37:2	C_40_H_72_O_6_K	−3.9
	701.5178	38:2	C_41_H_74_O_6_K	−5.6
	715.5337	39:2	C_42_H_76_O_6_K	−5.8
	731.5103	39:2	C_42_H_76_O_7_K	12.5
Ceramic vessel no. 5	673.4808	36:2	C_39_H_70_O_6_K	0.1
	687.4951	37:2	C_40_H_72_O_6_K	1.5
	701.5114	38:2	C_41_H_74_O_6_K	0.8
	715.5286	39:2	C_42_H_76_O_6_K	−0.7
	731.5007	39:2	C_42_H_76_O_7_K	22.1

CN: carbon number, equal to the total number of carbon atoms of the three fatty acid moieties; DB: number of double bonds; dtm: difference of measured mass from that calculated for a particular elemental composition.

**Table 2:** Results of the casein sandwich ELISA test and of the cattle β-lactoglobulin (βLG) competitive ELISA test used for the analysis of bottom soil layer samples taken from the studied vessels.

	Casein (ppm)	Evaluation	Cattle βLG (ppm)	Evaluation
Negative control	0.02	0	1.32	0
Positive control	0.2 *	+	0.021 **	+
Ceramic vessel no.4	0.36	+	1.81	0
Ceramic vessel no.5	0.52	+	0.044	+

+: positive, 0: negative, *: 0.5 mg/kg of casein standard, **: 10 mg/kg of ßLG.

## Supplementary Materials

The following are available online.
