# Supplementary material for: Determination of Milk Products in Ceramic Vessels of Corded Ware Culture from a Late Eneolithic Burial
Source: Molecules. 2018 Dec 7;23(12):3247. doi: 10.3390/molecules23123247 (PMC6321049; doi:10.3390/molecules23123247)
Supplement: Supplementary file 1 [file molecules-23-03247-s001.zip › molecules-398147-sup-final/Supplement-3.pdf]

Supplement 3.
Determination of Milk Products in Ceramic Vessels of Corded Ware Culture from a Late Eneolithic Burial

Lukáš Kučera [1], Jaroslav Peška [2], Pavel Fojtík [3], Petr Barták [1], Diana Sokolovská [1], Jaroslav Pavelka [4], Veronika Komárková [5], Jaromír Beneš [5], Lenka Polcerová [6], Miroslav Králík [6] and Petr Bednář [1,*]

[1] Regional Centre of Advanced Technologies and Materials, Department of Analytical Chemistry, Faculty of Science, Palacký University, 17. listopadu 12, 779 00 Olomouc, Czech Republic; lukas.kucera@upol.cz (L.K.); petr.bartak@upol.cz (P.Ba.); dia.sokolovska@gmail.com (D.S.); petr.bednar@upol.cz (P.Be.)

[2] Archaeological Centre Olomouc, U Hradiska 42/6, 779 00 Olomouc, Czech Republic; peska@ac-olomouc.cz (J.Pe.)

[3] Institute of Archaeological Heritage Brno, Kaloudova 1321/30, 614 00 Brno, Czech Republic; pavfojtik@seznam.cz (P.F.)

[4] Centre of Biology, Geoscience and Environmental Education, University of West Bohemia, Sedláčkova 15, 30614 Plzeň, Czech Republic; japetos@cbg.zcu.cz (J.Pa.)

[5] Laboratory of Archaeobotany and Palaeoecology, Faculty of Science, University of South Bohemia, Na Zlaté stoce 3, 370 05 České Budějovice, Czech Republic; verokomar@seznam.cz (V.K.); benes.jaromir@gmail.com (J.B.)

[6] Laboratory of Morphology and Forensic Anthropology (LaMorFA), Department of Anthropology, Faculty of Science, Masaryk University, Kotlářská 2, 611 37 Brno, Czech Republic; polcerova@seznam.cz (L.P.); mirekkralik@seznam.cz (M.K.)

* Correspondence: petr.bednar@upol.cz; Tel.: +42-0585-6344-03

[*] Corresponding author. Regional Centre of Advanced Technologies and Materials, Department of Analytical Chemistry, Faculty of Science, Palacký University, 17.listopadu 12, 779 00, Olomouc, Czech Republic
Tel.: +420585634403, E-mail address: petr.bednar@upol.cz

Supplement 3.

Burial content was cleaned, admixtures were excluded (mainly charcoals, weight 1.2 g) and osteological items were sieved through a sieve of 2 mm mesh size. Passed smallest pieces were weighed together on a digital scale. Remaining pieces were weighed and subsequently subjected by the following procedures. Visual assessment of skeletal fragments followed the recommendations published in literature [1-3]. Colour of the fragments was assessed by comparing with standard colour scales for cremated bones [4]. Skeletal fragments (pieces larger than 2 mm, Fig. 1S) were measured by means of original semiautomatic metric procedure developed by Polcerova [5].

A fragment of the pyramid of the temporal bone (*pars petrosa ossis temporalis*) with preserved internal acoustic canal (*meatus acusticus internus*) was subjected to the method for sex estimation developed by Norén et al [6]. Silicon casts of the canal and posterior side of the pyramid were made and the lateral angle between direction of the canal and posterior pyramid side was measured. The casting was repeated three times, on each cast lateral angle was measured five times and all resulting values were used for sex estimation. Skeletal elements were extremely fragmented and typical thermal cracks [3] were recorded. According to these features there are no doubts on the cremation origin of the changes. The majority of the fragments ranged in colour from light grey to white and were classified accordingly into the category of burning temperature 900 °C or higher. This corresponds to the grade V (chalky degree of burning, the bone is intensively white including on the broken surfaces, has a smooth surface and is hard and brittle) according to the method by Dokládal [2]. Total weight of the cremated remains was 2146.6 g (all 7973 fragments were measured). Maximum linear size of measured fragments ranged from 1.87 mm to 99.91 mm with an average of 13.27 mm (Fig. 2S).

Since two fragments of the same part of *protuberentia occipitalis interna (eminentia cruciformis)* and fragments of the same part of *margo supraorbitalis sin*. were found in the

* Corresponding author. Regional Centre of Advanced Technologies and Materials, Department of Analytical Chemistry, Faculty of Science, Palacký University, 17.listopadu 12, 779 00, Olomouc, Czech Republic
Tel.: +420585634403, E-mail address: petr.bednar@upol.cz

sample they cannot come from one individual. Therefore, the burial content represents remains of (at least) two human individuals. This finding is supported also by the total weight of the bone fragments (2,146.6 g), as it is recommended to consider more than one individual in burial content in cremated cases when total fragment weight exceeds 2,000 g [2,7]. According to the preserved third molar, the age at death of one of the individuals was at least 18 years of age [8].

Lateral angle values measured on the preserved pyramid of temporal bone ranged from 54° to 62° that is in the zone of variation occupied predominantly by females in the reference sample [6]. Specifically, 95% of confidence interval (CI) of mean for females was indicated as 46.25°–50.20° with standard deviation of 6.8° and range of 35.00°–65.00° and 95% CI of mean for males was indicated as 37.77°–40.90° with standard deviation of 6.4° and range of 25.00°–65.00° [6]. Therefore, one of the individuals included in the sample was more probably an adult female rather than adult male. The grave thus contained remains of one indefinable individual and probably woman’s remains.

* Corresponding author. Regional Centre of Advanced Technologies and Materials, Department of Analytical Chemistry, Faculty of Science, Palacký University, 17.listopadu 12, 779 00, Olomouc, Czech Republic
Tel.: +420585634403, E-mail address: petr.bednar@upol.cz

Fig. 1S. Cremated fragments of the analysed human skeleton(s); A: fragment of *os occipitale* with *protuberentia occipitalis interna*; B: *pars petrosa ossis temporalis* (measured *meatus acusticus internus*); C: fragment of *corpus vertebrae*; D: fragment of rib with *tuberculum costae*; Ea: *phalanx media* (*ossa manus*).

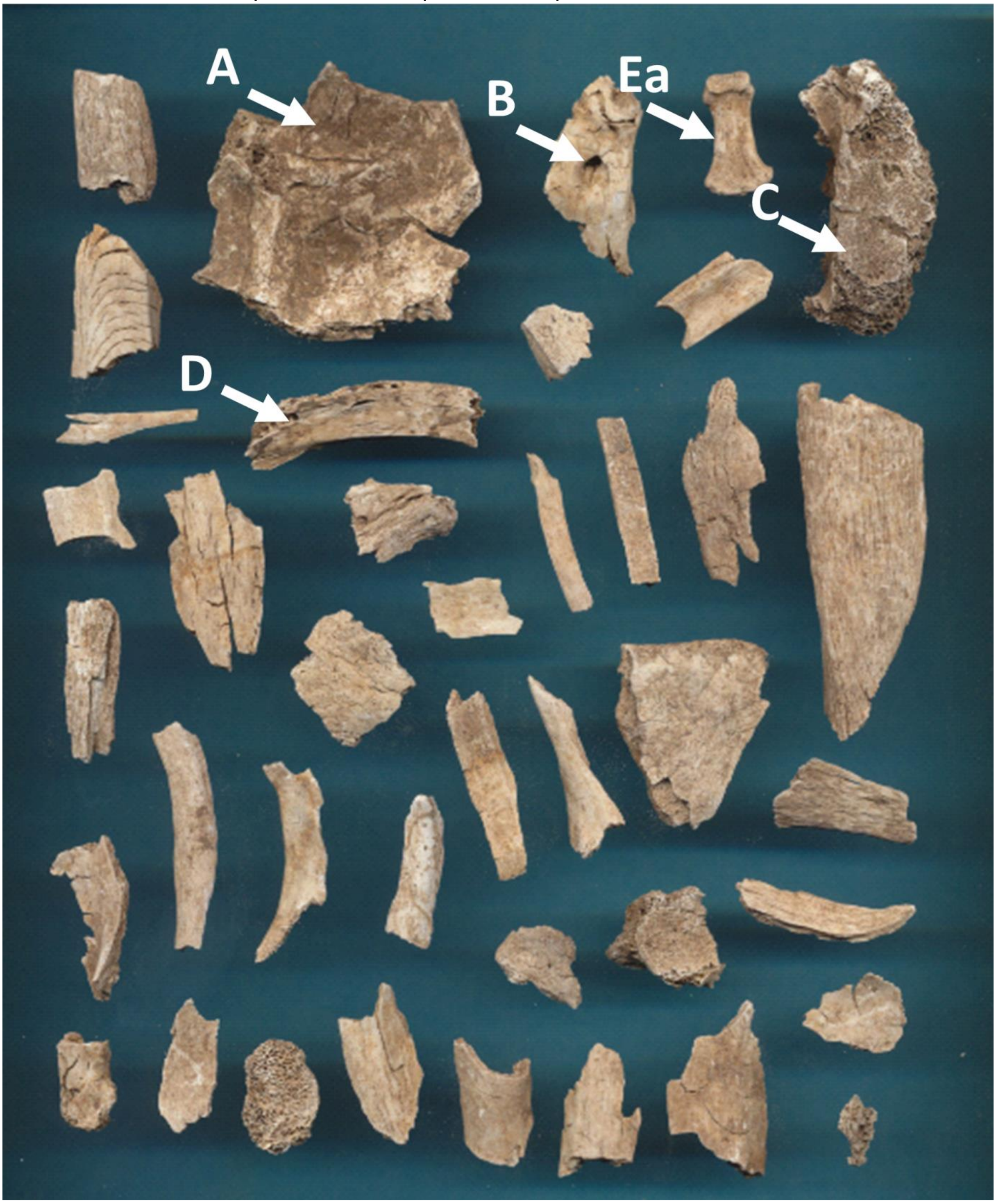

* Corresponding author. Regional Centre of Advanced Technologies and Materials, Department of Analytical Chemistry, Faculty of Science, Palacký University, 17.listopadu 12, 779 00, Olomouc, Czech Republic
Tel.: +420585634403, E-mail address: petr.bednar@upol.cz

Fig. 2S. Size of the bone fragments, log-normal distribution.

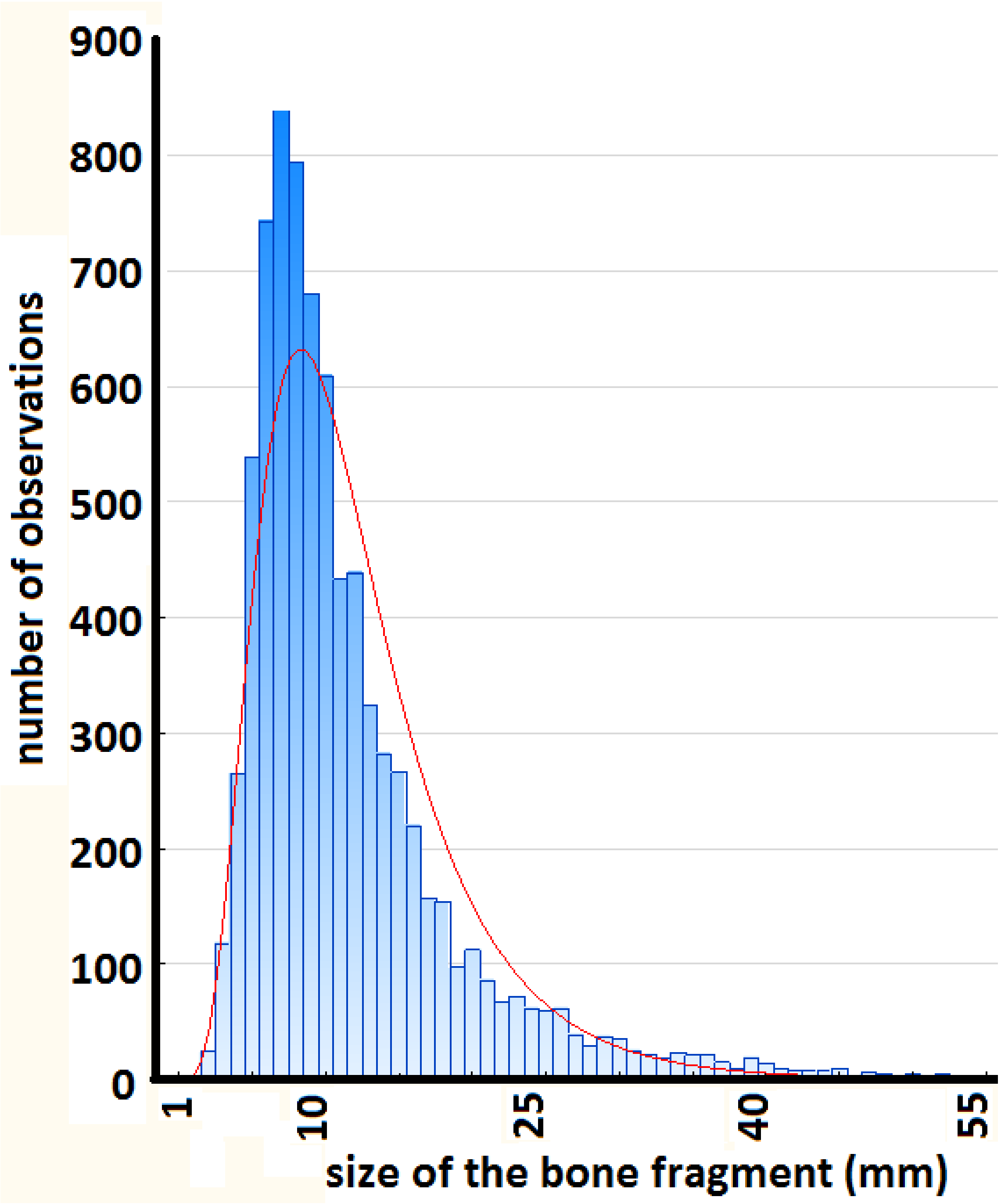

* Corresponding author. Regional Centre of Advanced Technologies and Materials, Department of Analytical Chemistry, Faculty of Science, Palacký University, 17.listopadu 12, 779 00, Olomouc, Czech Republic
Tel.: +420585634403, E-mail address: petr.bednar@upol.cz

Supplementary references

1. Chochol, J. Dosavadní výsledky anthropologického rozboru lužických žárových pohřbů z českých zemí. *Památky archeologické* **1955**, 49, 559–82.
2. Dokládal, M. Morfologie spálených kostí: význam pro identifikaci osob. Masaryk University, Faculty of Medicine, Brno 1999.
3. Symes, S.A.; Rainwater, C.W.; Chapman, E.N.; Gipson, D.R.; Piper, A.L. Patterned Thermal Destruction of Human Remains in a Forensic Setting, In The Analysis of Burned Human Remains, 15–54, Academic Press, San Diego 2008.
4. Walker, P.L.; Miller, K.W.P.; Richman, R. Time, Temperature, and Oxygen Availability: An Experimental Study of the Effects of Environmental Conditions on the Color and Organic Content of Cremated Bone, In The Analysis of Burned Human Remains (ed. S. A. Symes), 129–35, Academic Press, San Diego 2008.
5. Polcerová, L. Pars petrosa kosti spánkové v žárových hrobech, Bachelor Thesis, Masarykova univerzita, Ústav antropologie, Brno 2014.
6. Norén, A.; Lynnerup, N.; Czarnetzki, A.; Graw, M. Lateral Angle: A Method for Sexing Using the Petrous Bone. *Am. J. Phys. Anthropol.* **2005**, 128, 318–23.
7. Adams, B.J.; Byrd, J.E. Recovery, Analysis, and Identification of Commingled Human Remains. Humana press, New York 2008.
8. Lewis, M.J.; Senn, D.R. Dental Age Estimation, In Manual of Forensic Odontology 5th ed. (eds. D. R. Senn, and R. A. Weems), 211–55, CRC Press, Boca Raton 2013.

* Corresponding author. Regional Centre of Advanced Technologies and Materials, Department of Analytical Chemistry, Faculty of Science, Palacký University, 17.listopadu 12, 779 00, Olomouc, Czech Republic
Tel.: +420585634403, E-mail address: petr.bednar@upol.cz
