# Supplementary material for: Determination of Milk Products in Ceramic Vessels of Corded Ware Culture from a Late Eneolithic Burial
Source: Molecules. 2018 Dec 7;23(12):3247. doi: 10.3390/molecules23123247 (PMC6321049; doi:10.3390/molecules23123247)

Supplement 5.

Determination of Milk Products in Ceramic Vessels of Corded Ware Culture from a Late Eneolithic Burial

Lukáš Kučera [1], Jaroslav Peška [2], Pavel Fojtík [3], Petr Barták [1], Diana Sokolovská [1], Jaroslav Pavelka [4], Veronika Komárková [5], Jaromír Beneš [5], Lenka Polcerová [6], Miroslav Králík [6] and Petr Bednář [1,*]

[1] Regional Centre of Advanced Technologies and Materials, Department of Analytical Chemistry, Faculty of Science, Palacký University, 17. listopadu 12, 779 00 Olomouc, Czech Republic; lukas.kucera@upol.cz (L.K.); petr.bartak@upol.cz (P.Ba.); dia.sokolovska@gmail.com (D.S.); petr.bednar@upol.cz (P.Be.)

[2] Archaeological Centre Olomouc, U Hradiska 42/6, 779 00 Olomouc, Czech Republic; peska@ac-olomouc.cz (J.Pe.)

[3] Institute of Archaeological Heritage Brno, Kaloudova 1321/30, 614 00 Brno, Czech Republic; pavfojtik@seznam.cz (P.F.)

[4] Centre of Biology, Geoscience and Environmental Education, University of West Bohemia, Sedláčkova 15, 30614 Plzeň, Czech Republic; japetos@cbg.zcu.cz (J.Pa.)

[5] Laboratory of Archaeobotany and Palaeoecology, Faculty of Science, University of South Bohemia, Na Zlaté stoce 3, 370 05 České Budějovice, Czech Republic; verokomar@seznam.cz (V.K.); benes.jaromir@gmail.com (J.B.)

[6] Laboratory of Morphology and Forensic Anthropology (LaMorFA), Department of Anthropology, Faculty of Science, Masaryk University, Kotlářská 2, 611 37 Brno, Czech Republic; polcerova@seznam.cz (L.P.); mirekkralik@seznam.cz (M.K.)

* Correspondence: petr.bednar@upol.cz; Tel.: +42-0585-6344-03

Supplement 5. S-plots of acetone extracts of soil layers from ceramic vessel no. 4 and ceramic vessel no. 5.

S-plot (OPLS-DA) pareto; ceramic vessel no. 4

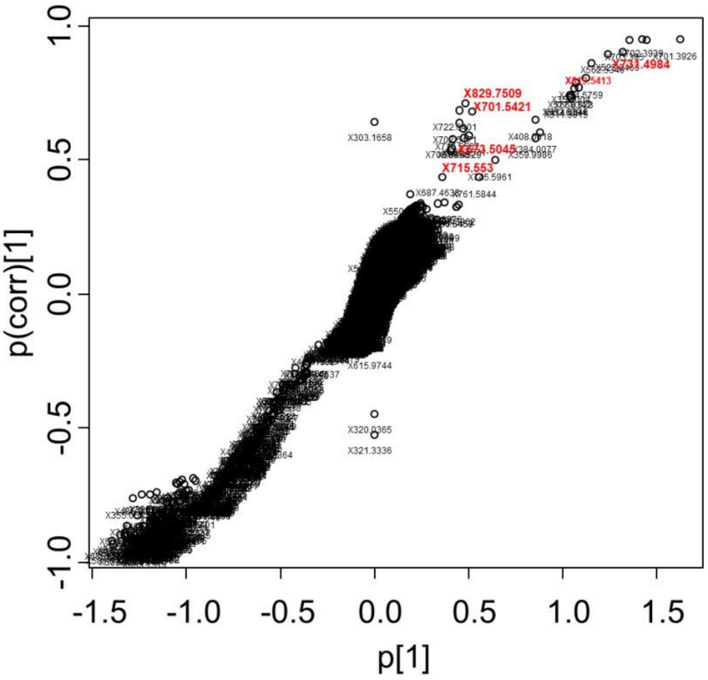

S-plot (OPLS-DA) pareto; ceramic vessel no. 5

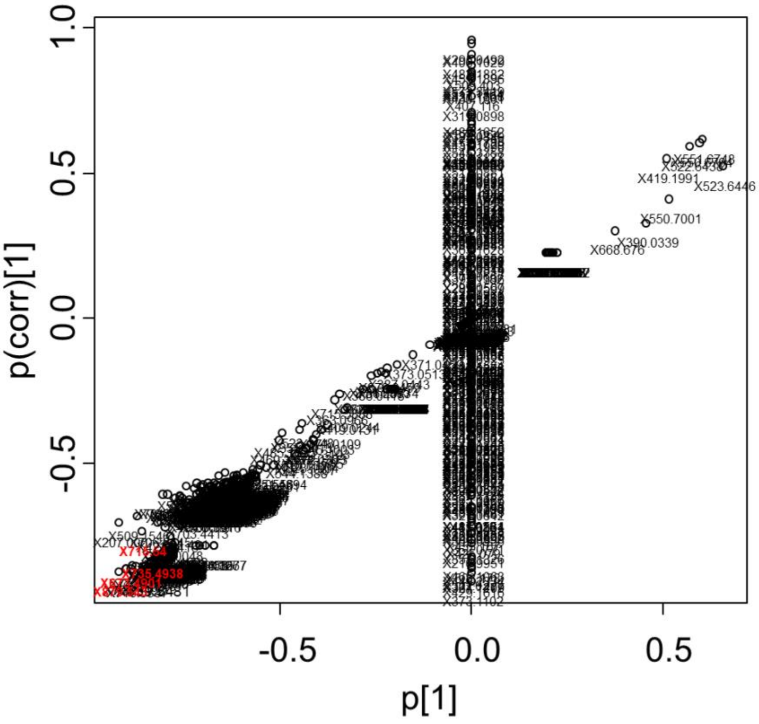

Supplement: Supplementary file 1 [file molecules-23-03247-s001.zip › molecules-398147-sup-final/Supplement-5.pdf]
